# Maxillary Arch Dimensions in Children with Unilateral Cleft Lip and Palate Receiving Alveolar Bone Grafting

**DOI:** 10.1177/10556656231188283

**Published:** 2023-07-13

**Authors:** Dewi M.E. van Stein Callenfels, Annemieke Bos, Ronald E.G. Jonkman

**Affiliations:** 1 Department of Orthodontics, 1192 Academic Centre for Dentistry Amsterdam (ACTA), Amsterdam, the Netherlands

**Keywords:** cleft lip and palate, alveolar bone graft, arch dimension, orthodontics

## Abstract

**Objective:**

To analyse the maxillary arch dimensions of children aged 9 and 12 with unilateral cleft lip and palate (UCLP) who received orthodontic treatment and secondary alveolar bone grafting.

**Design:**

This retrospective cohort study was performed on 30 patients with UCLP.

**Setting:**

All patients were treated at the Cleft Lip and Palate Centre, which is part of the University Medical Centre Amsterdam and Academic Centre for Dentistry Amsterdam, the Netherlands.

**Patients/Participants:**

Children with non-syndromic UCLP who received pre- and postsurgical orthodontics combined with secondary alveolar bone grafting between the ages of 9 and 12 years were included.

**Main Outcome Measures:**

Maxillary arch dimensions were assessed on 60 digitised dental casts with measurements of the intermolar widths, interpremolar widths, arch perimeters, arch lengths, arch widths, and palatal depths.

**Results:**

The results of a paired-samples *t*-test revealed a statistically significant increase (*P* < .05) in intermolar width 1, intermolar width 3, interpremolar width 1, arch perimeter, and arch width between the ages of 9 (T0) and 12 (T1). Intermolar width 2 and the palatal depth decreased statistically significantly between T0 and T1.

**Conclusions:**

Analysis of maxillary arch dimensions of children with UCLP indicates significant changes between 9 and 12 years of age. This suggests that orthodontic treatment and secondary alveolar bone grafting can be effective in improving maxillary arch dimensions. However, there is a need for collaborative research and data collection in order to provide sensible and evidence-based care to patients with cleft lip and palate.

## Introduction

Clefts can lead to various aesthetic, functional and dental problems among the affected patients, and surgical adjustment is considered an integral part to the current team approach for cleft treatment.^1,2^ The initial treatment focusses on both functional and aesthetic improvements by surgically closing the lip and soft palate which has a significant impact on the growth of the midface over the long term.^3–6^ During the mixed dentition stage, secondary alveolar bone grafting (SABG) is performed with autologous bone to repair the residuous cleft. This promotes the re-establishment of the dental arch continuity, stabilisation of the maxillary complex, and elimination of oronasal fistulae. As a result, this can improve speech, facilitate tooth eruption, and improve the facial appearance.^7,8^ Generally, orthodontic treatment prior to SABG is important for correcting misaligned incisors, repositioning displaced maxillary alveolar segments and expanding the maxillary arch if necessary.^9,10^ This also provides oral and maxillofacial surgeons with better access for placing the graft and closing the soft tissue.^9^ To achieve a successful outcome, SABG with pre- and postsurgical orthodontics is currently the standard of care for treatment of alveolar defects in patients with clefts who do not have sufficient arch width for direct placement of bone graft placement.^11^ Hence, the importance of orthodontists in planning, preparing for and following up on SABG procedures has been widely recognised.^10^

After a bone grafting procedure, new alveolar bone forms in approximately six weeks and becomes comparable to normal bone within two to seven months.^12,13^ Complete healing of the alveolar bone occurs at around 12 months.^14^ Three-dimensional imaging such as computed tomography is commonly used in treatment outcome verification and the evaluation of bone structures.^15^ Many studies have used three-dimensional imaging to evaluate changes in bone volume, facial skeletal structures and bony landmarks after alveolar bone grafting.^2,16–19^ Although no widely accepted standardised protocol exists for three-dimensional evaluation of SABG outcomes, the overall results obtained in these studies showed that while bone volume may decrease post-surgery, skeletal structures should remain stable.

For clinical relevance, it is important to understand how maxillary dental arch dimensions act over time. This aims to ensure that presurgical orthodontic treatment followed by SABG can effectively correct any maxillary dental arch issues in patients with unilateral cleft lip and palate. Moreover, it is important for clinicians to be knowledgeable about, and to be aware of changes in the maxillary arch dimensions in order to provide patients with UCLP a predictable and sensible treatment. Therefore, the aim of this study is to analyse the maxillary arch dimensions in children aged 9 and 12 with UCLP who received pre- and postsurgical orthodontics combined with SABG.

## Methods

This was a retrospective cohort study performed at the Academic Centre for Dentistry Amsterdam (ACTA). The study protocol was approved by the institutional review board of the centre (protocol number 2020252). In the present study, 81 consecutive patients with UCLP were recruited from patient files. All subjects were treated between 2010 and 2021 by the cleft lip and palate team of the University Medical Centre (UMC) Amsterdam and the ACTA and were enrolled upon an informed consent. Further selection was carried out according to the following inclusion criteria: (1) both sexes, (2) non-syndromic, (3) individuals who have undergone SABG between 9 and 12 years of age, (4) individuals who have undergone orthodontic treatment between 9 and 12 years of age, (5) inter-disciplinary treatment by the same cleft team, and (6) available digitised dental casts at approximately 9 and 12 years of age. The exclusion criteria were as follows: (1) other congenital malformations, (2) revision of alveolar bone grafting, and (3) missing digitised dental casts or casts of inadequate quality. Based on these eligibility criteria, 30 of 81 subjects were accepted in the study, and 51 subjects had to be excluded.

All 30 enrolled subjects were treated according to the treatment protocol of the cleft lip and palate team of the UMC Amsterdam and the ACTA and, corresponding to the Dutch national guidelines for the treatment and documentation of patients with clefts. No infant orthopaedics were carried out prior to the impressions taken at 9 years of age. After documentation at the age of 9, the presurgical orthodontic treatment consisted of either fixed appliances, removable expansion appliances or a combination of both to create a symmetrical maxillary arch form. After SABG, all subjects received subsequent orthodontic treatment for a period of 3 to 12 months. However, appliances were removed at least 12 months prior to impressions at the age of 12. The retention protocol consisted of a bonded wire retainer attached to the present upper incisors.

All orthodontic treatments were performed by experienced orthodontists from the cleft lip and palate team, employed at the ACTA. SABG surgeries were performed at the UMC Amsterdam by two experienced surgeons from the corresponding cleft lip and palate team. Both surgeons used the same surgical procedure, which included the division and closure of the nasoalveolar communication and the placement of an iliac crest or chin bone graft to fill the alveolar region of the cleft.

**Figure 1.**
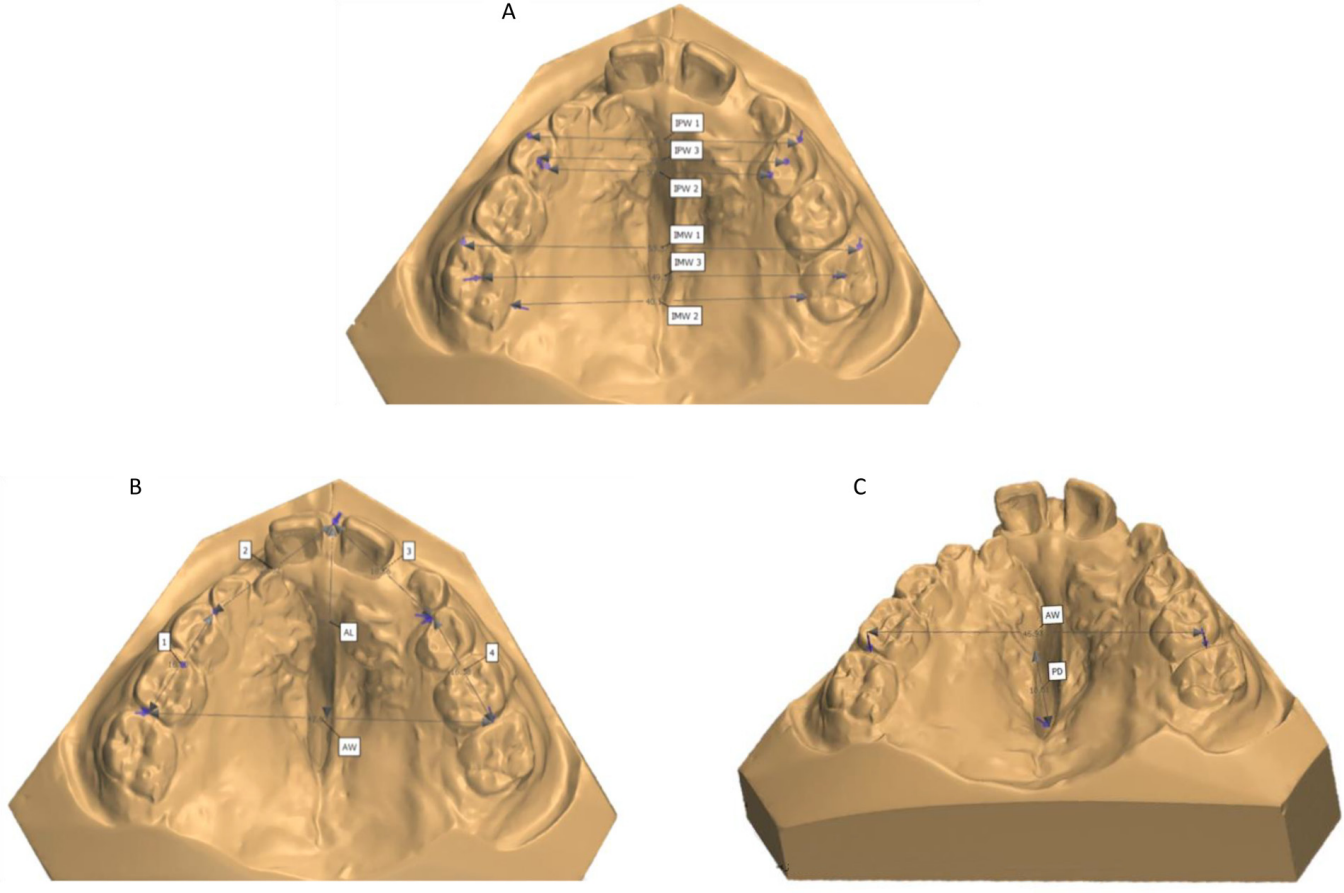
Measurements of the maxillary dental arch. (A) Intermolar width 1 (IMW1), intermolar width 2 (IMW2), intermolar width 3 (IMW3), interpremolar width 1 (IPW1), interpremolar width 2 (IPW2), and interpremolar width 3 (IPW3); (B) arch perimeter (AP = 1 + 2 + 3 + 4), arch length (AL), and arch width (AW); (C) palatal depth (PD) perpendicular to AW.

As part of the standardised treatment protocol, alginate impressions were taken at approximately 9 (T0) and 12 (T1) years of age and the dental casts were used for further evaluation. Subsequently, all dental casts were scanned using the 3Shape R700 scanner (3Shape A/S, Copenhagen, Denmark), which reproduces a three-dimensional digital image using laser beams projected over the casts in different directions. Then, 3D imaging of the scanned casts was performed using the 3Shape OrthoAnalyzer™ (3Shape A/S) programme, allowing frontal, lateral, posterior and occlusal visualisation. Next, reference points were independently marked on the digital casts by three examiners (DSC, TH and PE) to measure the intermolar widths (IMW1, IMW2, IMW3), interpremolar widths (IPW1, IPW2, IPW3), arch perimeter (AP), arch length (AL), arch width (AW) and palatal depth (PD) using the same software (Figure 1, Table 1). Each examiner independently performed the analyses, and all analyses were calibrated in advance. Examiner DSC was a third-year senior orthodontic resident, whereas examiners TH and PE were third-year dental students, both trained at the ACTA.

**Table 1.** Reference Points Used for Maxillary Arch Measurements.

Measurement	Description
Intermolar width	The distance in millimetre between the maxillary first permanent molars, measured at three different reference points: IMW1; mesiobuccal cusp IMW2; palatal fissure IMW3; central occlusal fissure
Interpremolar width	The distance in millimetre between the maxillary first premolars/deciduous first molars, measured at three different reference points; IPW1; mesiobuccal cusp IPW2; palatal cusp IPW3; central occlusal fissure
Arch perimeter (AP)	The perimeter of the maxillary arch in millimetre as the sum of: The mesial contact point of the maxillary first permanent molar to the mesial contact point of the maxillary first premolar/deciduous first molar The mesial contact point of the maxillary first premolar/deciduous first molar to the midpoint between the maxillary central incisors The midpoint between the maxillary central incisors to the mesial contact point of the maxillary first premolar/deciduous first molar The mesial contact point of the maxillary first premolar/deciduous first molar to the mesial contact point of the maxillary first molar
Arch length (AL)	The distance in millimetre of the line between the midpoint of the central incisors perpendicular to the line of the arch width
Arch width (AW)	The distance in millimetre between the mesial contact points of the maxillary first permanent molars
Palatal depth (PD)	The distance in millimetre between the intersection point of the arch width and arch length to the palate

In total, 20% of the sample was analysed again after a three-week interval under similar conditions to determine the measurements’ reliability. Inter- and intra-examiner agreements were calculated using the intra-class correlation coefficient (ICC).

Descriptive statistics, including means and standard deviations, were calculated for all measurements. Additionally, the ethnic backgrounds of all subjects were noted as follows: 12 subjects of Asian origin, 11 subjects of Caucasian origin, and 7 of mixed or other origin. One-way ANOVA determined statistically significant differences could not be observed for arch dimensions. Ethnicity therefore has not affected our results and the three groups were considered comparable. Maxillary arch dimensions were compared between children aged 9 and 12 using a paired-samples *t*-test. The level of significance was set at 5% (*P* < .05). All statistical analyses were performed in SPSS Statistics version 17.0 for Windows (SPSS Inc., Chicago, IL, USA).

## Results

In total, 60 digitised maxillary casts of 30 patients with UCLP were included. The sample characteristics are summarised in Table 2, with 22 male and 8 female subjects. The mean age was 8.11 years (SD = 0.290) at the time of the first dental impression (T0)**,** and 12.0 years (SD = 0.805) at the second dental impression (T1). Closure of the hard palate using bone grafting was performed at a mean age of 10.0 years (SD = 0.675).

**Table 2.** Sample Characteristics.

Variable	
Gender: male/female	22/8
Side of cleft: left/right	21/9
Pre-surgical orthodontic treatment: fixed appliances/ expansion plate + fixed appliances/ expansion plate	18/10/2
Age: 9-year casts (years.months)	Mean 8.11 (SD 0.290)
Age: 12-year casts (years.months)	Mean 12.0 (SD 0.805)
Age at SABG (years.months)	Mean 10.0 (SD 0.675)

The inter-rater reliability for the measurements was assessed using intraclass correlation coefficient (ICC) scores, which were found to be excellent with all scores above 0.9 (Table 3). Since the inter-rater agreement was excellent, the measurements performed by researcher DSC were used for further evaluation. The intra-rater agreement scores were also calculated using the ICCs for intermolar and interpremolar widths, arch perimeters, arch lengths, arch widths**,** and palatal depths with all scores being excellent and above 0.9 (Table 3).

**Table 3.** Mean ICC Scores for Intra-Rater Agreement and Inter-Rater Agreement for all Measurements.

	IMW1	IMW2	IMW3	IPW1	IPW2	IPW3	AP	AL	AW	PD
Intra-rater	0.999	0.999	0.999	0.999	0.999	0.999	0.998	0.993	0.995	0.994
Inter-rater	0.959	0.964	0.959	0.991	0.958	0.974	0.922	0.969	0.953	0.969

The results obtained for the maxillary arch dimension at 9 (T0) and 12 (T1) years of age are presented in Table 4. The results obtained from the paired-samples *t-*test revealed a statistically significant difference (*P* < .05) in intermolar width 1 (IMW1), intermolar width 2 (IMW2), intermolar width 3 (IMW3), interpremolar width 1 (IPW1), arch perimeter (AP), arch width (AW), and palatal depth (PD) between both ages. With respect to the molar-to-molar distance, IMW1 and IMW3 showed a statistically significant increase, while IMW2 statistically significantly decreased between T0 and T1. All interpremolar widths increased between T0 and T1, with only IPW1 showing a statistically significant change. Moreover, the arch perimeters and arch widths also showed a statistically significant increase. Arch lengths increased between 9 and 12 years of age, but this difference was not statistically significant. Finally, the palatal depths statistically significantly decreased.

**Table 4.** Mean Maxillary Arch Dimensions at 9 (T0) and 12 Years of age (T1) and Their Comparison Using the Paired-Samples *t*-Test.

	Mean T0 (mm)	95% CI	Mean T1 (mm)	95% CI	Mean change T1-T0 (mm)	95% CI	*p*
IMW1	50.17 (SD 3.54)	48.97-51.81	51.15 (SD 3.32)	50.14-52.76	0.99 (SD 1.53)	0.39-1.58	0.002*
IMW2	37.23 (SD 3.50)	36.45-38.87	36.45 (SD 3.17)	35.40-38.02	-0.78 (SD 1.54)	-1.38−0.19	0.012*
IMW3	45.73 (SD 3.44)	44.60-47.36	46.33 (SD 3.14)	45.44-47.87	0.60 (SD 1.38)	0.06-1.13	0.030*
IPW1	36.78 (SD 3.54)	35.44-38.45	38.95 (SD 4.46)	37.63-41.12	2.17 (SD 4.04)	0.57-3.77	0.010*
IPW2	29.00 (SD 3.14)	27.55-30.25	30.07 (SD 3.81)	28.32-31.61	1.08 (SD 3.10)	-0.18-2.33	0.089
IPW3	32.98 (SD 3.82)	31.29-34.61	34.17 (SD 3.83)	32.44-35.77	1.19 (SD 3.65)	-0.28-2.66	0.109
AP	68.00 (SD 5.24)	66.40-70.80	69.48 (SD 5.72)	67.99-72.59	1.48 (SD 3.64)	0.12-2.84	0.034*
AL	24.74 (SD 2.73)	23.89-26.25	25.29 (SD 2.73)	24.55-26.85	0.56 (SD 1.94)	-0.17-1.28	0.127
AW	42.53 (SD 3.50)	41.44-44.14	43.27 (SD 3.33)	42.13-44.70	0.74 (SD 1.90)	0.03-1.45	0.041*
PD	13.72 (SD 3.06)	12.21-14.99	10.75 (SD 1.83)	9.84-11.55	-2.97 (SD 2.55)	-3.92−2.02	0.000*

*P < .05.

## Discussion

This retrospective cohort study aimed to describe changes in maxillary arch dimensions in 30 non-syndromic children with UCLP at two different ages. The results revealed an overall increase in the maxillary arch dimensions between the ages of 9 and 12, except for the intermolar width measured from the palatal fissures and palatal depth. Statistically significant increases were found in IMW1, IMW3, IPW1, arch perimeter, and arch width between both ages. These changes in intermolar widths may be a result of the orthodontically prepared arch form, stabilised and supported by the newly formed alveolar bone after SABG. Notably, a statistically significant decrease in IMW2 was observed, which could be explained by derotation of the upper first permanent molars in a non-cleft group. However, in subjects with cleft in which the palatally placed maxillary arch segments require correction, an increase in IMW2 would be expected. Regarding the interpremolar width, measurements increased between T0 and T1, yet only IPW1 statistically significantly changed. It must be taken into account that there is a difference in anatomy between the maxillary deciduous first molar and maxillary first premolar.^20,21^ Finally, the alveolar bone grafting procedure ensures a significant decrease in arch depth.

Previous studies have analysed maxillary arch dimensions in children with clefts and compared them to children without.^22–26^ However, there is a lack of longitudinal data on dental arch measurements at this important age at the time of alveolar bone grafting, although some attempts for comparison have been made. For example, in this study, the mean intermolar width measured between the buccal cusps of the first molars for children aged 9 was 50.17 mm. This finding contrasts with the results of a previous study by Generali et al.^24^ on 19 children with UCLP, who reported a smaller intermolar width of 44.9 mm at the same age. Nevertheless, the arch length found in this study for children aged 9 was 24.74 mm, whereas a larger value of 27.16 mm was found in the sample of Athanasiou et al.^22^ at the same age. At the age of 12 years, mean arch length was 25.29 mm in this study, which is smaller than the value of 27.44 mm found in the sample of Athanasiou et al.^22^ However, comparing these findings is challenging due to the different cleft types, surgical and orthodontic protocols, ages of the sample and measurement methods used.

Overall, previous studies suggest that maxillary arch dimensions in children with clefts are significantly smaller than the normal dimensions and that at the age of 12 years the intermolar widths can reach values close to the normal. When the results of this study are compared to the arch width measurement data of Moyers et al.^20^ in children without clefts, the following can be observed. The mean IMW3 at 9 years of age in this study was 45.73 mm (SD = 3.44), compared to slightly smaller intermolar widths of 43.1-44.5 and 42.4-43.5 mm for males and females without clefts, respectively. At the age of 12 years, the IMW3 values in this study (46.33 mm, SD = 3.14) were comparable but slightly larger than the 45.3 and 44.6 mm found for males and females without clefts, respectively. Hence, it seems that the mean intermolar width in this study is quite comparable to the one in children without clefts. Notably, the sample in this study was not divided into more groups on the basis of sex, as this would not provide significant information. Moreover, no significant association has been found between sex and intermolar width in patients with clefts, although sex significantly influences the size of the maxillary arches in subjects without clefts.^27^ Therefore, the presence of the cleft itself seems to have a larger influence than sex on the morphological characteristics of the maxillary arch.^28^

Generally, retrospective data is often associated with confounders of selection bias and initial-state differences. Due to limitations in sample sizes, time constraints, and ethical considerations, relying solely on randomised controlled trials to evaluate the effectiveness of protocols and procedures for patients with clefts is not always feasible. It is noted that the sample of this study was of limited size, and there may be other factors influencing the effectiveness of orthodontic treatment and SABG, such as the severity of the cleft, the timing and technique of surgical interventions, and the quality of postoperative care. Future research should utilise a standardised treatment protocol, including data collection at specific ages and comparing results with a control group, in order to increase statistical power and enable inter-centre comparisons. In this, digitising casts offers significant advantages, such as improved storage and diagnostic versatility, and offers a high level of accuracy and reproducibility in measurement.^29^ Despite the limitations in this study, the findings have important clinical implications for the management of children with CLP. Yet, this renders continuing research on patients with clefts is of great interest in order to gain knowledge, evaluate and predict treatment, and improve care for patients with special treatment needs.

## Conclusion

Analysis of the maxillary arch dimensions of children with UCLP who received orthodontic treatment and SABG indicates significant changes between 9 and 12 years of age.This suggests that orthodontic treatment and SABG can be effective in improving maxillary arch dimensions. However, there is a need for continuing collaborative research and data collection in order to provide sensible and evidence-based care for patients with cleft lip and palate.
